# Bioinformatics-driven identification of prognostic biomarkers in kidney renal clear cell carcinoma

**DOI:** 10.3389/fneph.2024.1349859

**Published:** 2024-04-04

**Authors:** Varinder Madhav Verma, Sanjeev Puri, Veena Puri

**Affiliations:** ^1^ Centre for Systems Biology and Bioinformatics, Panjab University, Chandigarh, India; ^2^ Biotechnology University Institute of Engineering and Technology (UIET), Panjab University, Chandigarh, India

**Keywords:** renal cell carcinoma, prognostic biomarkers, bioinformatics analysis, TCGA-KIRC, Qiagen IPA, survival analysis, cox proportional-hazards model

## Abstract

Renal cell carcinoma (RCC), particularly the clear cell subtype (ccRCC), poses a significant global health concern due to its increasing prevalence and resistance to conventional therapies. Early detection of ccRCC remains challenging, resulting in poor patient survival rates. In this study, we employed a bioinformatic approach to identify potential prognostic biomarkers for kidney renal clear cell carcinoma (KIRC). By analyzing RNA sequencing data from the TCGA-KIRC project, differentially expressed genes (DEGs) associated with ccRCC were identified. Pathway analysis utilizing the Qiagen Ingenuity Pathway Analysis (IPA) tool elucidated key pathways and genes involved in ccRCC dysregulation. Prognostic value assessment was conducted through survival analysis, including Cox univariate proportional hazards (PH) modeling and Kaplan–Meier plotting. This analysis unveiled several promising biomarkers, such as *MMP9*, *PIK3R6*, *IFNG*, and *PGF*, exhibiting significant associations with overall survival and relapse-free survival in ccRCC patients. Cox multivariate PH analysis, considering gene expression and age at diagnosis, further confirmed the prognostic potential of *MMP9*, *IFNG*, and *PGF* genes. These findings enhance our understanding of ccRCC and provide valuable insights into potential prognostic biomarkers that can aid healthcare professionals in risk stratification and treatment decision-making. The study also establishes a foundation for future research, validation, and clinical translation of the identified prognostic biomarkers, paving the way for personalized approaches in the management of KIRC.

## 1 Introduction

Kidney cancer is the seventh most prevalent cancer in men and the 10th most common cancer in women, accounting for 5% and 3% of all adult malignancies in men and women, respectively. The average age for diagnosis is 60 years old, with a notable gender disparity existing for receiving a diagnosis. Renal cell carcinoma (RCC) is a sneaky neoplasm that accounts for 2% of all cancer diagnoses and fatalities globally and is expected to become more prevalent (1). More developed regions like Europe and North America have a higher incidence of kidney cancer than less developed ones like Africa and South America. An estimated 372,000 persons worldwide are affected by kidney cancer each year (2).

Of all primary renal neoplasms, 80% to 85% are RCCs, which develop within the renal cortex whereas 8% of cancers are transitional cell carcinomas, which develop in the renal pelvis. RCCs, often known as kidney cancers, are a class of histologically identified tumors that can be characterized by several genetic alterations. Renal cancer has several diverse subtypes, each of which has a unique histology, distinctive genetic and molecular changes, and hence unique response to treatment. The three main RCC subtypes (with ≥5% incidence), which account for 75%, 15%, and 5% of RCCs, are clear cell RCC (ccRCC), papillary RCC (pRCC), and chromophobe RCC (chRCC), respectively (2). Other subtypes of RCC like transitional cell carcinoma, Wilms tumor, and renal sarcoma have ≤1% total incidence and are very rare. Approximately 30% of individuals experience recurrence following the complete removal of the initial tumor. All RCC subtypes are largely unresponsive to conventional chemotherapy and radiation therapy (3).

The pathologic stage and nuclear grade largely determine the prognosis for ccRCC (4). Clear cell tumors feature well-defined cell boundaries and translucent, empty (water-clear) cytoplasm. Variable levels of lipid and glycogen can be seen in the cytoplasm. Radial or partial nephrectomy is the main form of treatment. Despite having mixed results, chemotherapy and immunotherapy are utilized on individuals with metastatic illness. Its clinical symptoms usually appear in later stages, and due to its high morbidity and mortality, early detection and accurate prognosis are critical for improving patient treatment and outcomes, but there are several challenges associated with these goals.

The widespread adoption of RNA-seq has heightened the demand for bioinformatics skills and computational resources to analyze large-scale biological data efficiently and unraveling molecular complexities in genomics research. For this study, RNA-seq data were analyzed. It initially involves isolation of total RNA from target tissues or cells, which then entails building DNA libraries followed by sequencing with a next-generation sequencing approach. RNA-seq data have several advantages over other transcriptome methods, like RNA-seq offers a better resolution and dynamic range, making it more sensitive and can reliably estimate gene expression at a wider range of levels. By RNA-seq data, one can find novel transcripts, alternative splicing events, and non-coding RNAs, and detect known transcripts and quantify these. It also does not possess any bias in the selection of probes, in contrast to microarrays, which depend on pre-designed probes. The availability of such type of data in publicly accessible databases, such as The Cancer Genome Atlas (TCGA) (https://www.cancer.gov/tcga) and Genotype-Tissue Expression (GTEx) databases (https://www.gtexportal.org/home/), has provided researchers with comprehensive datasets for various cancer types, including ccRCC.

A massive amount of research on various aspects of ccRCC has been done (5, 6), including analysis of the cross-model features of ccRCC for prognosis prediction (7) or indulgence of artificial intelligence for studying the pathomics and genomics of RCC (8). However, in this study, we have taken a computational approach to analyzing the DEGs and carrying out the network analysis and pathways involved in ccRCC. Here, we have identified potential prognostic biomarkers for ccRCC using data from the TCGA by selecting the RNA-seq data under the project ID, TCGA-KIRC (kidney renal clear cell carcinoma). We uncovered differentially expressed genes (DEGs) associated with ccRCC using the R programming language. These identified DEGs were analyzed for pathways enrichment using the Qiagen Ingenuity Pathway Analysis (IPA) tool to gain insights into the dysregulation of key pathways involved in ccRCC. We selected the top 10 candidate genes from the gene interaction networks of the identified DEGs. These genes were further investigated by gene expression profiling and survival analysis. The identified candidate genes including the overall survival (OS) and relapse-free survival (RFS) along the Cox multivariate analysis led to a set of three genes as potential biomarkers for ccRCC in the present study.

## 2 Methods

**Figure 1:**
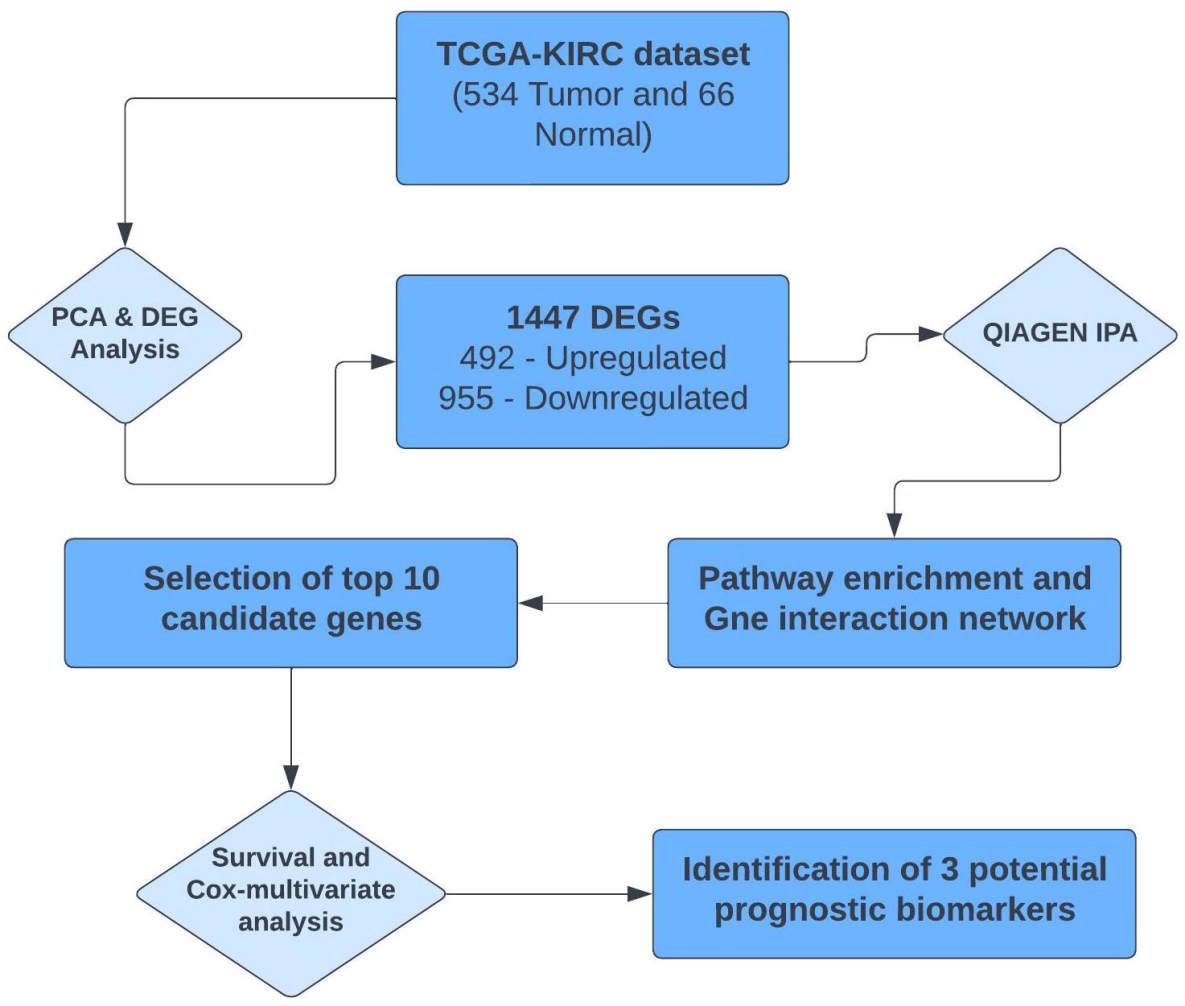
Summarized workflow of carrying out RNA-seq analysis of the TCGA-KIRC project.

The complete summarized workflow adopted for the RNA-seq analysis of the TCGA-KIRC data has been described in **Figure 1**. Each method used is described in detail below.

### 2.1 Data screening from the TCGA database

In this study, we obtained the RNA-seq data for KIRC from the widely recognized TCGA database. Leveraging the TCGA biolinks R package, the dataset, which encompassed a total of 613 samples, was downloaded. Within this dataset, 541 samples represented tumorous tissue, providing valuable insights into the gene expression profiles associated with KIRC. The remaining 72 samples were designated as normal, serving as a vital reference for understanding the gene expression patterns in non-cancerous kidney tissue.

### 2.2 Data pre-processing and identification of DEGs

Data pre-processing and identification of DEGs were conducted through a systematic pipeline, employing various essential steps facilitated by the edgeR package of Bioconductor (9). The data were imported, organized, filtered, and normalized to ensure robustness and accuracy in subsequent analyses.

To address potential false positives, a *p*-value correction was carried out, utilizing the Benjamini–Hochberg method. A significance threshold of adjusted *p*-value less than 0.05 was strictly applied to identify the top genes exhibiting significant differential expression. Additionally, a log2FC value of ±2 was utilized to discern upregulated and downregulated genes within our dataset. Subsequently, the visualization of DEGs was achieved through the implementation of the ggplot2 package, generating a volcano plot.

### 2.3 Pathway analysis of differentially expressed genes and the selection of candidate genes

Pathway analysis of the DEGs was performed using the widely acclaimed IPA tool (version – 90348151) by Qiagen. The DEGs were analyzed by Qiagen IPA for its “Core Analysis,” where the default settings were used, with a significance threshold of *p*-value < 0.05 and a log2FC cutoff of ±2. This rigorous analysis allowed us to uncover the crucial biological pathways significantly affected by the DEGs, providing invaluable insights into the underlying molecular mechanisms. From the list of pathways obtained, the pathways with a threshold equivalent to the *p*-value of 0.05 were considered.

To gain a comprehensive understanding of the gene interactions within each pathway, the “My Pathways” module of IPA was explored for constructing gene interaction networks to decipher the intricate relationships between the genes. This module also incorporated the “Molecule Activity Predictor (MAP),” which utilizes a vast knowledge base derived from manual curation of scientific literature to predict gene/molecule activation or inhibition. The selection of candidate genes followed this; here, the top 10 DEGs that were involved in most of the pathways were considered for downstream analysis.

### 2.4 Gene expression profiles and survival analysis

The gene expression profiles were analyzed using the GEPIA2 web server, a powerful online tool designed to explore and visualize RNA-seq data from two prominent projects, namely, TCGA and the GTEx database (10). The GEPIA2 web server offers a comprehensive suite of functionalities, including the generation of box plots to depict the expression patterns of selected candidate genes.

To assess the prognostic significance of the identified candidate genes, survival analysis including the Kaplan–Meier plots for the OS and RFS was conducted using the same GEPIA2 platform. This feature of GEPIA2 utilizes the Cox proportional hazards model, a widely accepted statistical method for survival analysis, to calculate the hazard ratio (HR).

Cox multivariate statistical analysis was used to assess the simultaneous effects of multiple variables on survival outcomes in cancer research. The gene expression data related to the candidate genes and clinical data were downloaded from the Xena Functional Genomics Explorer by the University of California Santa Cruz (https://xenabrowser.net/) (11). The analysis was performed using the survival package of R software, where the covariates include the demographic details of patients, i.e., age at initial diagnosis. The *p*-value for this analysis was set at 0.05.

## 3 Results

### 3.1 Data processing and identification of differentially expressed genes

**Figure 2:**
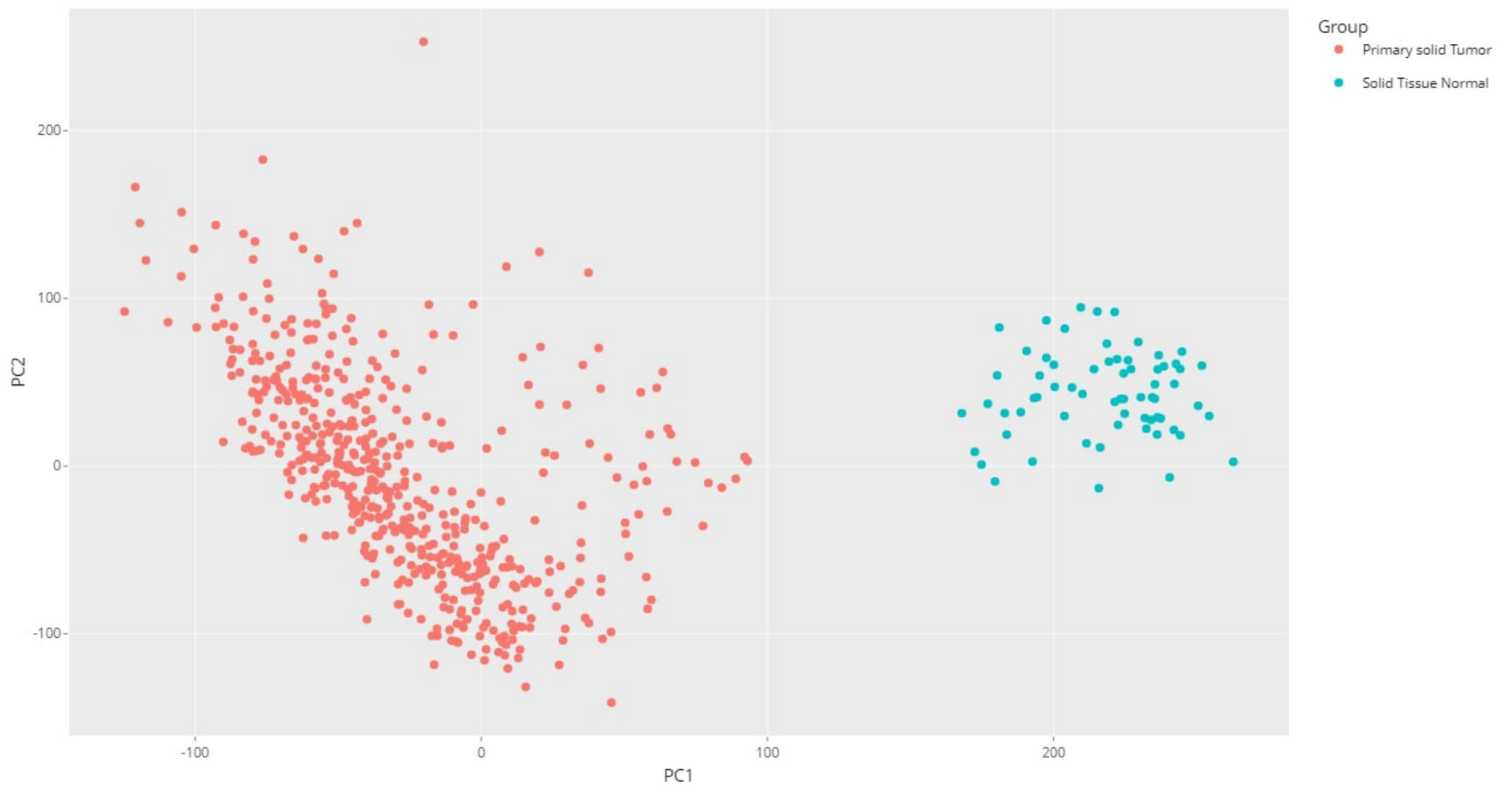
PCA plot for the KIRC dataset using R programming language (after processing the raw data).

**Figure 3:**
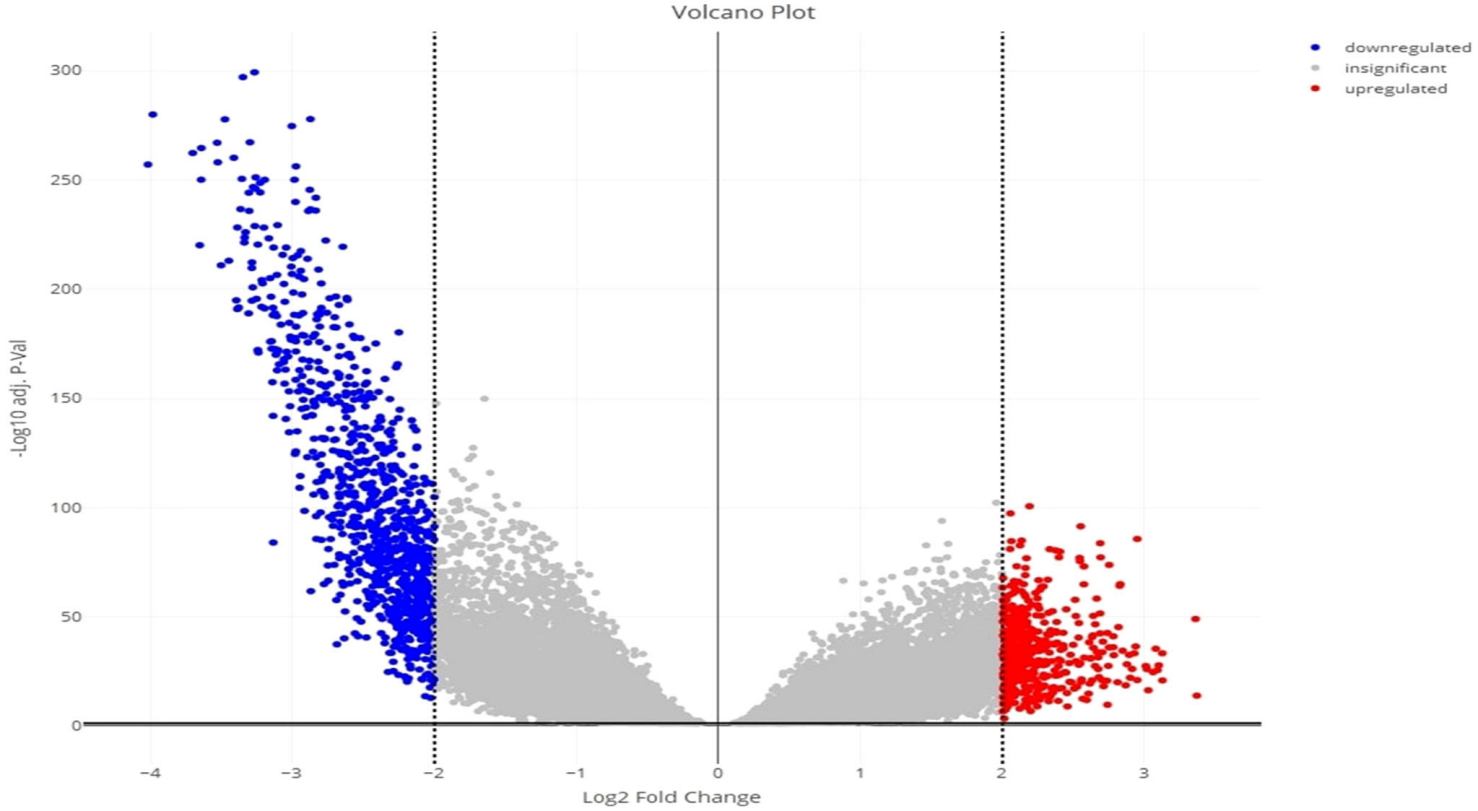
Volcano plot for the identified DEGs, showing up- and downregulated genes in red and blue color, respectively.

Principal component analysis (PCA) of the downloaded dataset led us to the identification of outliers that were filtered from the dataset using the R programming language. A total of 13 samples were filtered from a set of 613 samples as these were outliers in their respective groups. PCA obtained after processing the data clearly demarcated the normal (66) and tumor (534) samples, making a total count of 600 samples (**Figure 2**). Using the edgeR-limma pipeline, we identified 1,447 DEGs including 492 upregulated genes and 955 downregulated genes in the TCGA-KIRC dataset, after the FDR analysis using the Benjamini–Hochberg method. This has been visualized via the Volcano plot shown in **Figure 3**, where the red color represents upregulated genes, while the blue color represents downregulated genes. (A full list of up- and downregulated genes has been provided in the **Supplementary Data Sheet** “DEGs list”).

### 3.2 Pathway analysis of DEGs using IPA

The core analysis in Qiagen IPA resulted in the 19 pathways that were associated with statistically significant DEGs, as seen in **Table 1**. Some of the highly downregulated pathways include CREB Signaling in Neurons, Phagosome Formation, and the S100 Family Signaling Pathway, while pathways like the Pathogen-Induced Cytokine Storm Signaling Pathway, IL-17 Signaling, and IL-4 Signaling pathway are upregulated. Dysregulation of all these pathways has been found in the progression of different types of cancers.

**Table 1:** IPA pathway analysis result. Top 19 pathways involved in the DEGs of KIRC.

S. No.	Ingenuity Canonical Pathways	Molecules	*z*-score
**1**	Pathogen-Induced Cytokine Storm Signaling Pathway	26	3.138
**2**	Role of Osteoblasts in Rheumatoid Arthritis Signaling Pathway	21	2.4
**3**	Wound Healing Signaling Pathway	20	1.342
**4**	IL-17 Signaling	17	1.213
**5**	IL-4 Signaling	16	1
**6**	Neuroinflammation Signaling Pathway	25	0.894
**7**	Ovarian Cancer Signaling	15	0.333
**8**	LPS/IL-1 Mediated Inhibition of RXR Function	17	0
**9**	ERK/MAPK Signaling	17	0
**10**	Natural Killer Cell Signaling	17	-0.728
**11**	Neurovascular Coupling Signaling Pathway	18	-1.213
**12**	Chaperone Mediated Autophagy Signaling Pathway	19	-1.606
**13**	Breast Cancer Regulation by Stathmin1	41	-1.718
**14**	FAK Signaling	50	-1.98
**15**	Neuroprotective Role of THOP1 in Alzheimer’s Disease	18	-2.138
**16**	G-Protein Coupled Receptor Signaling	45	-2.236
**17**	S100 Family Signaling Pathway	55	-2.292
**18**	Phagosome Formation	52	-2.496
**19**	CREB Signaling in Neurons	42	-3.086

**Figure 4:**
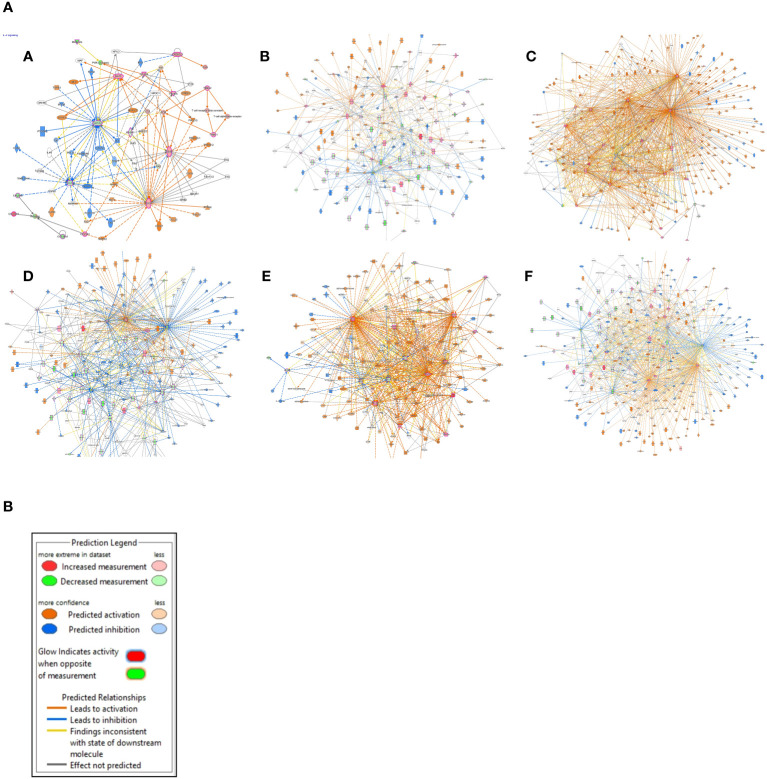
**(A)** Gene interaction networks. (A) IL-4 signaling, (B) phagosome formation, (C) pathogen-induced cytokine storm signaling, (D) CREB signaling in neurons, (E) IL-17 signaling pathway, and (F) S100 family signaling pathway. **(B)** The color scheme used for the nodes that represent our dataset, as well as the ones that are predicted using the “Molecule Activity Predictor (MAP)” tool from the “My Pathway” module of IPA.

After the pathways analysis, gene networks were constructed to determine how the identified DEGs in KIRC interact with each other and the genes/molecules present in the default pathway taken from the Ingenuity knowledge base. The gene interaction networks for the above-mentioned pathways (**Figure 4A**) and legends are shown in **Figure 4B**. (The gene interaction networks for the rest of the pathways are available in the **Supplementary Image Data**).

### 3.3 Selection of candidate genes

The data obtained from the IPA was subject to rigorous analysis to identify the most crucial genes driving the dysregulation of numerous pathways. To ensure a robust selection, we focused on the top 10 DEGs deciphered from pathway enrichment criteria that might have pivotal roles in the pathogenesis of KIRC. *PIK3C2G, PIK3R5, PIK3R6, MMP9, VEGFA, CREB3L3, IFNG, PGF*, *GRM1*, and *RASD1* were identified as the top 10 DEGs. These selected genes were contributing important functions in at least seven distinct pathways (detailed in the **Supplementary Data Sheet** “Top 10 candidate genes”). The involvement of these genes in critical cellular pathways sheds light on their potential as key drivers of KIRC pathomechanistic progression (12–14).

### 3.4 Gene expression profiles and survival analysis

**Figure 5:**
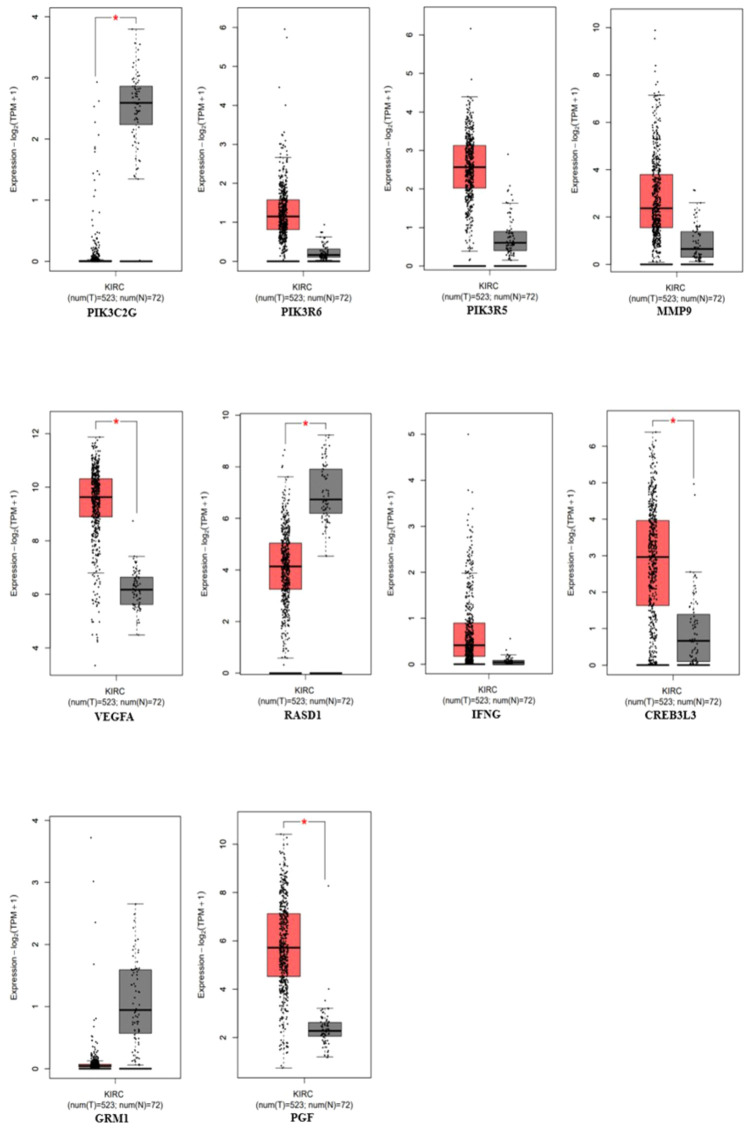
Gene expression profiles of the top 10 genes using the expression analysis module in the GEPIA-2 tool (*PIK3C3G, PIK3R6, PIK3R5, MMP9, VEGFA, RASD1, IFNG, CREB3L3, GRM1*, and *PGF*), *p*-value < 0.05 (red = tumor, gray = normal).

**Figure 6:**
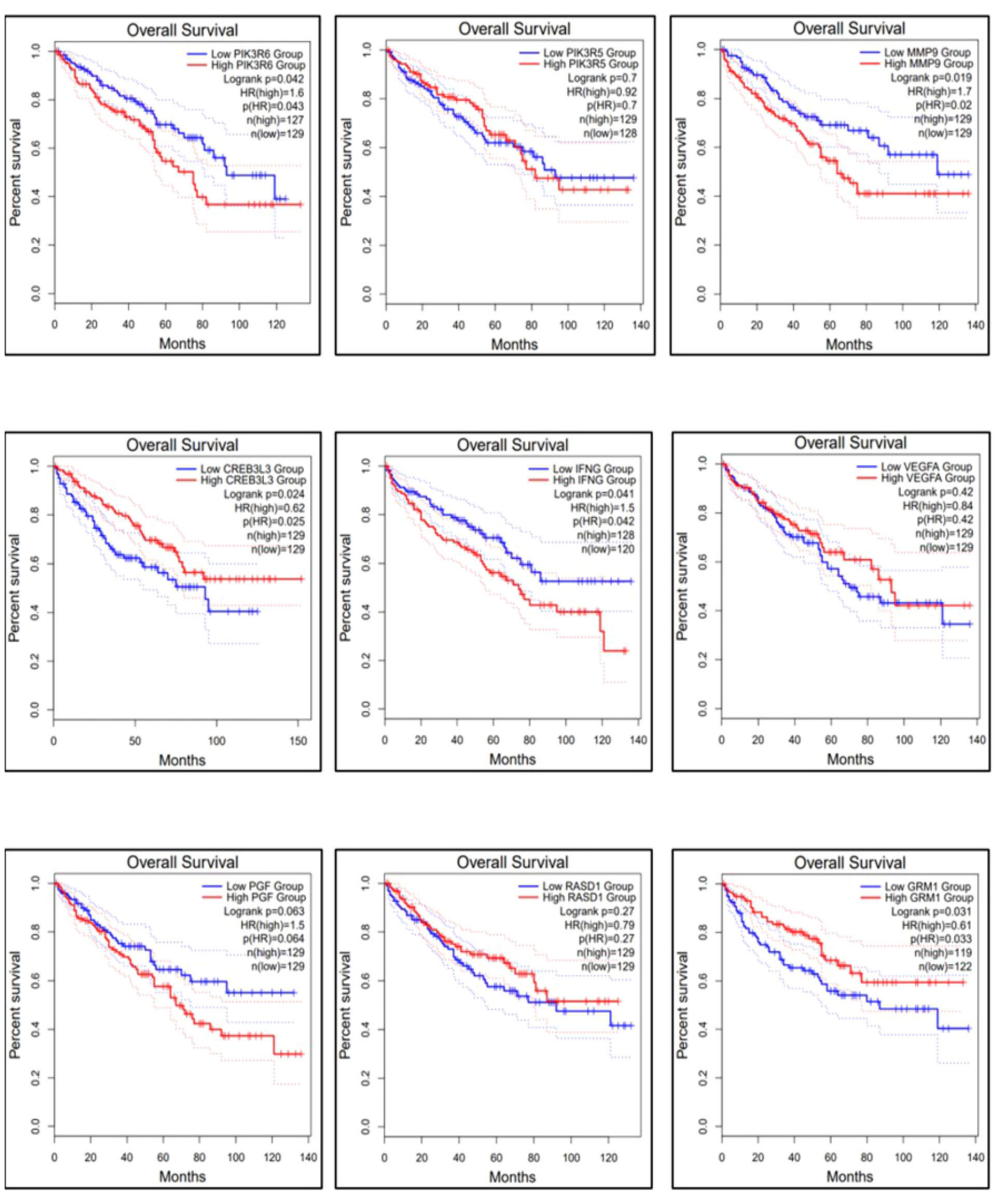
Overall survival of the top 9 genes using the survival analysis module in GEPIA-2. The dysregulation of genes *MMP9, PIK3R6*, and *IFNG* is involved in the poor prognosis of KIRC.

**Figure 7:**
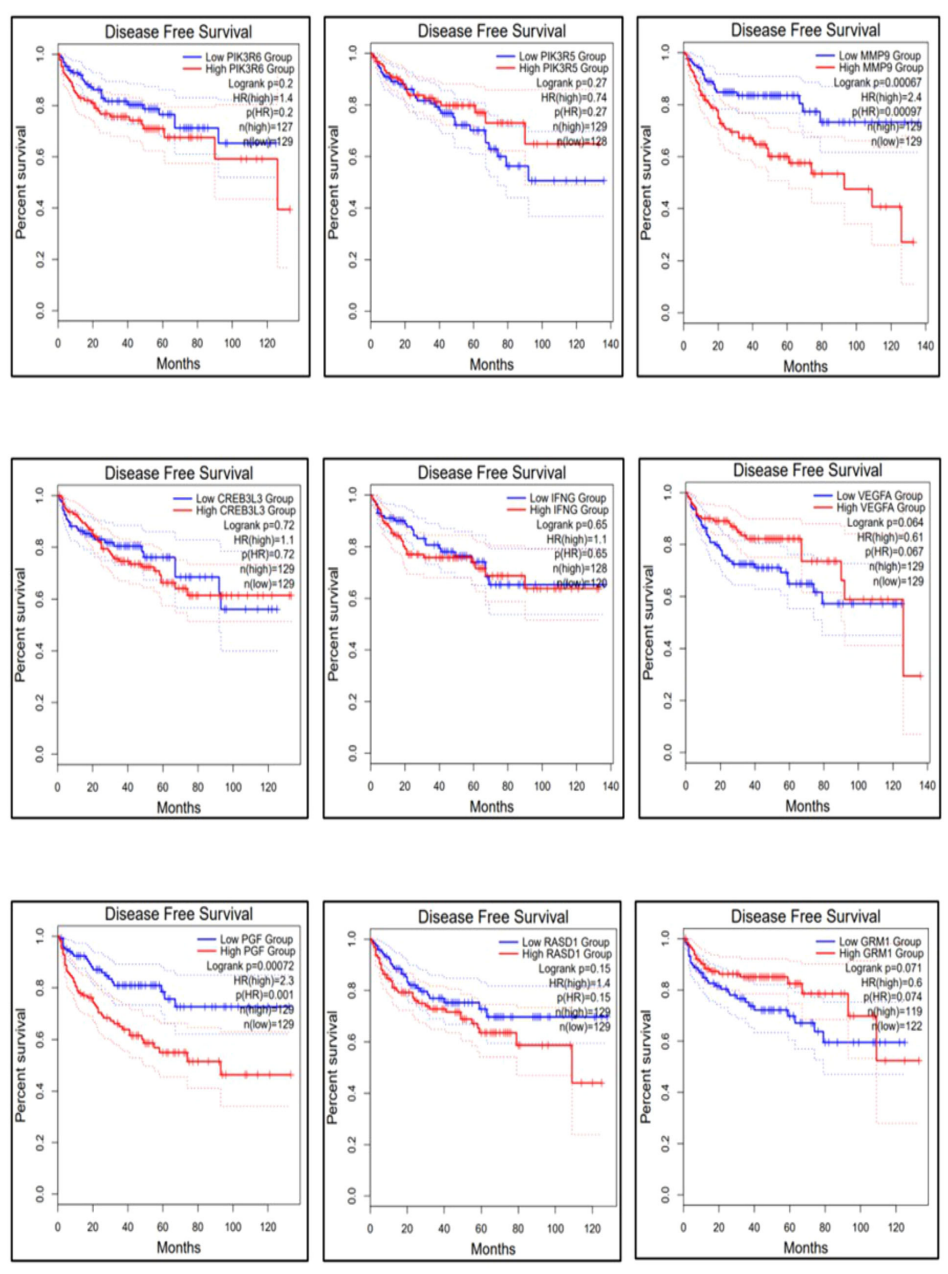
Relapse-free survival of the top 9 genes using the survival analysis module in GEPIA-2. The dysregulation of genes *MMP9* and *PGF* is highly responsible for the recurrence of KIRC.

The gene expression profiles of the top 10 candidate genes in the form of boxplots for both tumor and normal samples are shown in **Figure 5**. To evaluate their prognostic significance, comprehensive survival analyses were conducted for OS and RFS. The Kaplan–Meier plots were generated, accompanied by HRs and their respective 95% confidence interval (CI) ranges. Among the 10 candidate genes, *MMP9* exhibited an HR of 1.7 (log-rank *p* = 0.019), *PIK3R6* showed an HR of 1.6 (log-rank *p* = 0.042), *CREB3L3* showed an HR of 0.62 (log-rank *p* = 0.024), and *IFNG* presented an HR of 1.5 (log-rank *p* = 0.041) in association with OS (**Figure 6**). Additionally, *MMP9* demonstrated a substantial HR of 2.4 (log-rank *p* = 0.00067), and *PGF* showed a remarkable HR of 2.3 (log-rank *p* = 0.00072) in association with RFS (**Figure 7**).

Based on these findings, *MMP9*, *PIK3R6*, *IFNG*, and *PGF* emerged as potential candidates for prognostic biomarkers in KIRC. For a concise overview, a summary of the survival analysis, along with pertinent statistical data, is presented in **Table 2**. This highlights the key findings of the survival analyses and underscores the clinical significance of the identified genes as potential prognostic markers in KIRC. It was observed that the expression of *PIK3C2G* was not significant in KIRC (**Figure 5**), prompting its exclusion from further analysis.

**Table 2:** Survival analysis results, * sign represents the potential prognostic biomarkers, i.e., *MMP9, PIK3R6*, and *IFNG* in the case of OS; *MMP9* and *PGF* in the case of RFS.

Gene	Overall Survival (OS)		Relapse-Free Survival (RFS)	
	HR	Log-rank *p*	HR	Log-rank *p*
MMP9* (↑)	1.7	0.019	2.4	0.00067
PIK3R6* (↑)	1.6	0.042	1.4	0.2
IFNG* (↑)	1.5	0.041	1.1	0.65
PGF* (↑)	1.5	0.063	2.3	0.00072
***PIK3R5*** (↑)	0.92	0.7	0.74	0.27
***VEGFA* (↑)**	0.84	0.42	0.61	0.064
***RASD1* (**↓)	0.79	0.27	1.4	0.15
***CREB3L3*** (↑)	0.62	0.024	1.1	0.72
***GRM1* (**↓)	0.61	0.031	0.6	0.071
***PIK3C2G* (**↓)	-NA-	-NA-	-NA-	-NA-

(↑) = Upregulated genes; (**↓**) = Downregulated genes. NA: Not Applicable.

**Figure 8:**
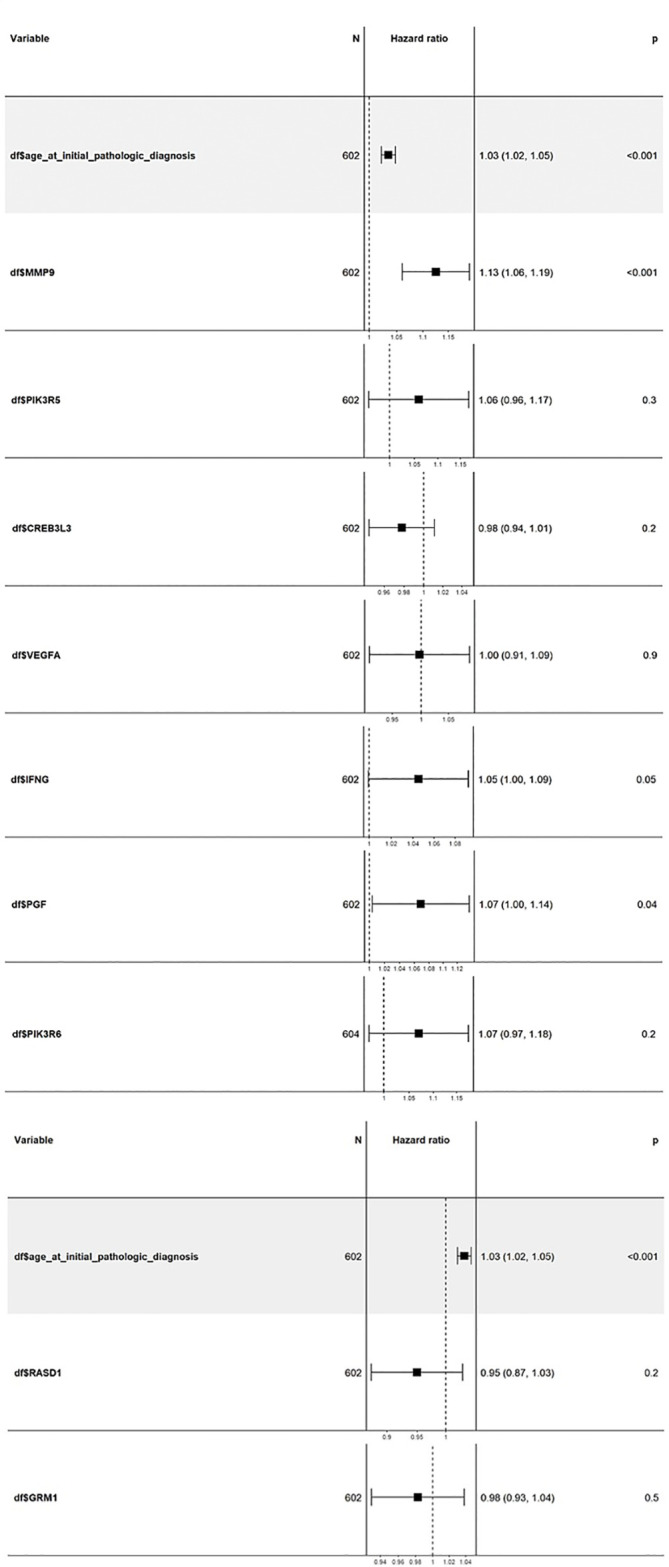
Cox multivariate analysis of the top 9 candidate genes using the “coxph” package in the Bioconductor. The genes *MMP9, IFNG*, and *PGF* show high HR values with a significance of 0.05 or less. ccRCC, Clear cell renal cell carcinoma; DEGs, Differentially expressed genes; HR, Hazard ratio; IPA, Ingenuity Pathway Analysis; KIRC, Kidney renal clear cell carcinoma; OS, Overall survival; PCA, Principal component analysis; RCC, Renal cell carcinoma; RFS, Relapse-free survival; TCGA, The Cancer Genome Atlas.

Cox multivariate analysis was employed to comprehensively assess the prognostic significance of all nine candidate genes (**Figure 8**). The covariates age, gender, and tumor stage were considered for this method. No significant results were obtained in the case of gender and tumor stage; however, the results for age as a covariate were significant. The covariate “age at initial diagnosis” exhibited an HR of 1.03, with a remarkable 95% CI ranging from 1.02 to 1.05. The highly significant *p*-value of <0.001 indicates that, for each additional year of age at diagnosis, the risk of adverse outcomes in KIRC patients escalates by a factor of 1.03 or 3%. This trend consistently persisted throughout the analysis, underscoring the critical influence of age on patient outcomes. Among the nine genes, *MMP9*, *IFNG*, and *PGF* displayed HR values greater than 1, implying their association with an increased risk of adverse outcomes in KIRC patients. Furthermore, these three genes demonstrated statistical significance with *p*-values of 0.05 or less, reinforcing their potential as robust prognostic indicators. Conversely, the gene *PIK3R6* exhibited weak statistical significance and was consequently excluded from further consideration.

## 4 Discussion

ccRCC is the most prevalent subtype of RCC, with a 5-year survival rate of less than 10% (15). However, post-treatment survival with Lenvatinib and Pembrolizumab showed an increased median OS of 53.7 months (16). ccRCC is very rare to be diagnosed at an early stage, hence lowering the survival rate of patients. Therefore, more research for early diagnosis of this disease and more precise treatment needs to be provided, lowering the death rate.

Pathway analysis from IPA showed that the pathways highly downregulated have been reported in Phagosome Formation, CREB Signaling in Neurons, and S100 Family Signaling Pathway, while pathways related to immune profiling like the Pathogen-Induced Cytokine Storm Signaling Pathway, IL-17 Signaling, and IL-4 Signaling pathway are upregulated by our identified 492 upregulated genes and 955 downregulated DEGs. Cyclic adenosine monophosphate (cAMP) is an important second messenger; the proliferation, differentiation, death, and immunological responses of cells are just a few of the biological processes that cAMP can control. Any dysregulation or modification of cAMP signaling, i.e., CREB signaling, may result in immunological dysfunction, illness, or cancer as well as cell metabolic disorders (14). It is important to note that phagosomes are cellular compartments responsible for engulfing and digesting foreign particles. The downregulation of the Phagosome formation pathway in the context of cancer may have several implications, such as immune evasion and affecting tumor-associated macrophages, creating an immunosuppressive environment. *In vivo* and *in vitro* experimentation on tumor-associated markers and their phagocytic function have elucidated multiple outcomes that are plausible in the tumor microenvironment (17). The proteins in the S100 family perform a variety of intracellular and extracellular functions. They work with a variety of receptors and signal transducers to control pathways that govern inflammation, cell differentiation, proliferation, and apoptosis (12). Dysregulation of the cytokine signaling pathway brought on by pathogens is a major factor in the initiation and spread of cancer. There is an abnormal cytokine and growth factor expression in a variety of malignancies, including lymphomas and solid tumors, including IL-6 and IL-10 (13, 18). These improperly functioning cytokines can serve as autocrine growth factors that encourage the survival, multiplication, and invasion of tumor cells (19). There is evidence that IL-17 mediates the release of additional proinflammatory cytokines and chemokines, particularly from the smooth muscle and pulmonary epithelium (20).

The results obtained from the survival analysis showed that the genes *MMP9*, *PIK3R6*, and *IFNG* are associated with decreased OS, while *MMP9* and *PGF* are associated with increased chances of recurrence (RFS) of KIRC. These genes had an HR value ≥ 1.5 and a log-rank *p*-value of less than 0.05. Moreover, the Cox multivariate analysis of these candidate genes where the age at initial diagnosis was considered as a covariate results in the exclusion of the *PIK3R6* gene due to its low statistical significance. Hence, the genes *MMP9*, *IFNG*, and *PGF* are identified as potential prognostic biomarkers for the KIRC.

*MMPs* are fascinating genes associated with the development of cancer, functional angiogenesis enhancement, invasion, metastasis, and evasion of immune surveillance. These genes are typically elevated in cancer, and murine studies suggest a role in tumor-associated tissue remodeling (21). According to the research carried out from a pan-cancer perspective, it has been shown that the prognostic value of *MMPs* is limited to ccRCC (22). As seen in the results, the *MMP9* gene, which is upregulated in this dataset, had HR values of 1.7 and 2.4 in the OS and RFS, respectively, with high significance rates.

*PIK3R6* is highly upregulated and is involved in the dysregulation of 14 different pathways. As per the survival analysis results obtained, this gene resulted in poor prognosis, as the high expression of *PIK3R6* decreases the OS rate. Moreover, in a recent study, the relationship between dysregulated lipid metabolism genes (LMGs) and ccRCC progression was investigated. Here, they established a novel risk score model, according to which the *PIK3R6* gene was predicted to have a poor prognosis, higher immune pathway activation, and cancer development (23). However, later in this analysis, *PIK3R6* was removed from the list due to its low significance rate.

*PGF* is a member of the *VEGFA* family and is highly involved in cell proliferation and growth factor activity (24). It has been seen that the plasma levels of PGF can be employed as a standalone prognostic factor for RCC due to their substantial connection with clinical characteristics and VEGF levels (25). The high HR value of 2.3 and the log-rank *p*-value of 0.0007 make this gene highly significant and a potential prognostic marker for KIRC after MMP9.

*IFNG* is known for assisting in the prevention of neoplastic disease (26) and is a potent activator of macrophages. Inhibiting growth, making tumor cells more susceptible to death, increasing the expression of MHC class I and class II, and promoting antitumor immune activity are all effects of *IFNG*. Shorter lung cancer survival has been associated with decreased *IFNG* serum levels (27). Nevertheless, the opposite is observed in KIRC, where high expression of *IFNG*, instead of suppressing the tumor, results in lowering the survival rate by almost 1.5 times as compared to its lower expression. This contradiction might be context-dependent, and further research to elucidate the underlying mechanism is needed.

Apart from these four genes, *CREB3L3*, which is highly upregulated in the KIRC dataset, shows that an increase in its expression increases the OS of KIRC patients; a similar profiling for this gene has been reported in the Human Proteome atlas. According to previous studies, the genes of the *CREB* family behave differently, most of which have a tumor-suppressing nature like the gene *CREB3L3*, which is identified as a low-risk gene having high expression in the case of bladder cancer (28). It was also found that high expression of *CREB3L3* helps in the bad prognosis of gastric cancer (29). It might be possible that this gene, being a low-risk gene, prevents KIRC progression.

## 5 Conclusion

In conclusion, this comprehensive research delves into the identification of potential prognostic biomarkers for ccRCC using transcriptome data analysis. The results shed light on the dysregulation of critical pathways associated with immune response, angiogenesis, and cytokine signaling in ccRCC. Through survival analysis, *MMP9* emerged as a robust prognostic marker for both OS and RFS, with high expression levels correlating with adverse outcomes. This is followed by *PIK3R6* and *IFNG* whose high expression levels had poor survival outcomes. *PGF* also showed promising potential as a prognostic marker for recurrence. The Cox multivariate analysis provided additional support to the prognostic significance of *MMP9*, *IFNG*, and *PGF*, considering the age at diagnosis as a covariate. However, *IFNG’s* role appeared context-dependent, as *IFNG*, being a tumor-suppressing gene, promotes tumor progression as observed in the results. Therefore, further research is needed to elucidate the underlying mechanisms.

These findings contribute significantly to our understanding of ccRCC and have important clinical implications. The identification of prognostic biomarkers like *MMP9* and *PGF* can aid in risk stratification and treatment decision-making, potentially leading to more precise and personalized approaches for patients with ccRCC. In the quest for early detection and more effective treatments, this study paves the way for continued investigation, validation, and potential clinical translation of these identified prognostic biomarkers.

## Data availability statement

Publicly available datasets were analyzed in this study. This data can be found here: TCGA-KIRC through the GDC Data Portal.

## Ethics statement

Ethical approval was not required for the study involving humans in accordance with the local legislation and institutional requirements. Written informed consent to participate in this study was not required from the participants or the participants’ legal guardians/next of kin in accordance with the national legislation and the institutional requirements.

## Author contributions

VV: Formal analysis, Investigation, Methodology, Software, Validation, Writing – review & editing, Data curation, Writing – original draft. SP: Formal analysis, Investigation, Methodology, Software, Writing – review & editing, Conceptualization, Funding acquisition, Resources, Supervision, Visualization. VP: Conceptualization, Formal analysis, Funding acquisition, Investigation, Methodology, Software, Supervision, Visualization, Writing – review & editing, Validation.
