# Supplementary figures and images for: Bioinformatics-driven identification of prognostic biomarkers in kidney renal clear cell carcinoma
Source: Front Nephrol. 2024 Apr 4;4:1349859. doi: 10.3389/fneph.2024.1349859 (PMC11024385; doi:10.3389/fneph.2024.1349859)

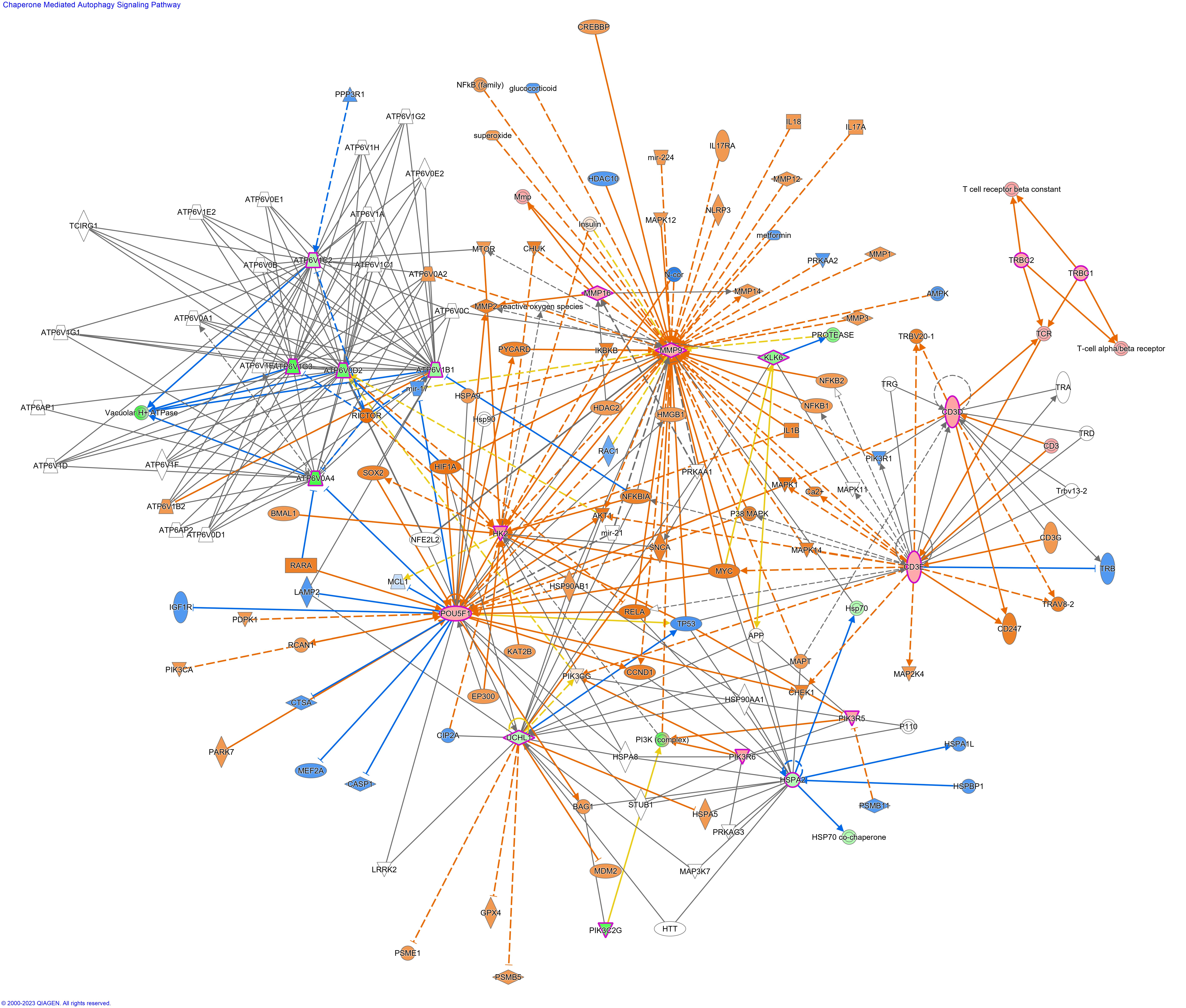

Supplement: Supplementary file 3 [file Image_2.jpeg]

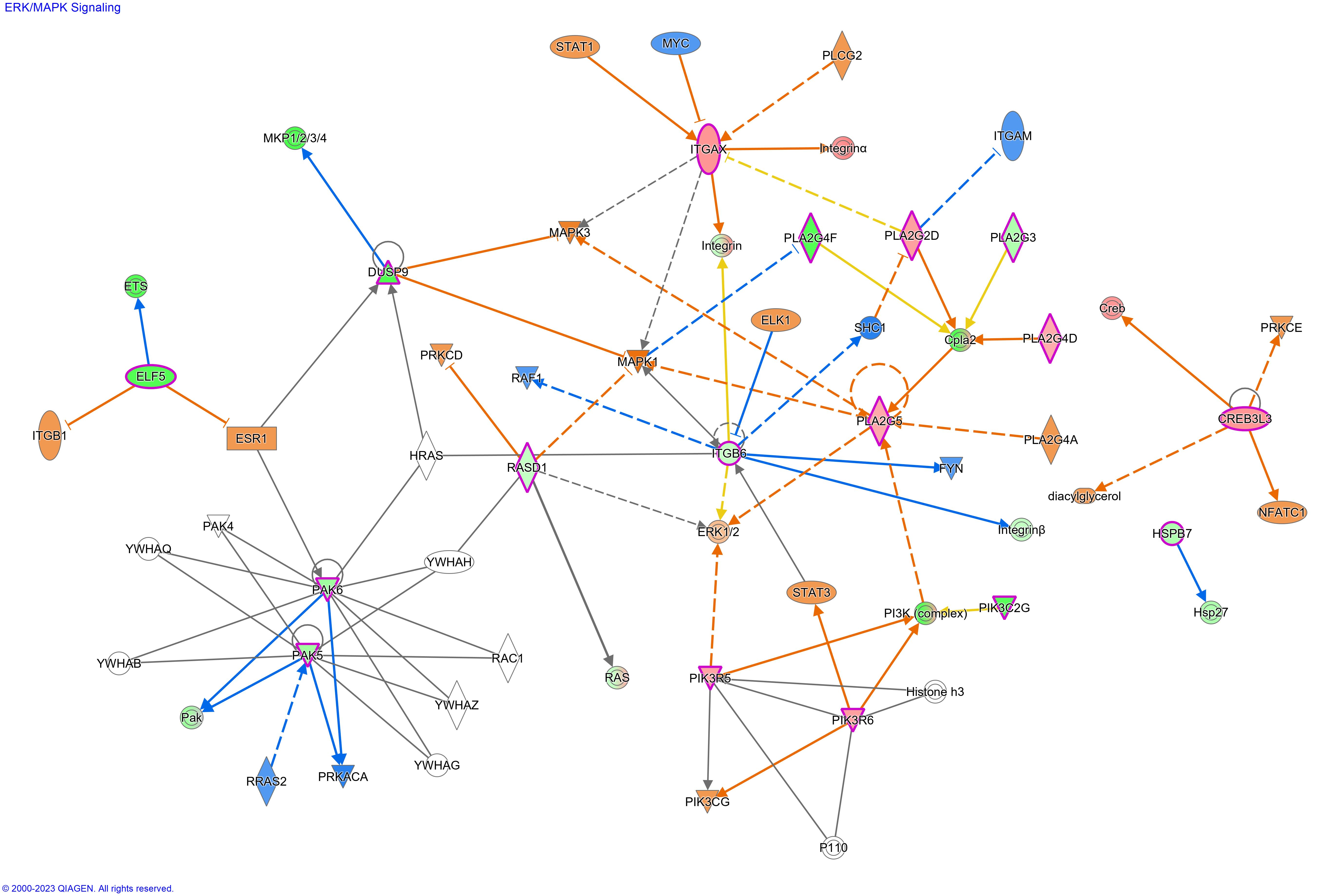

Supplement: Supplementary file 5 [file Image_4.jpeg]

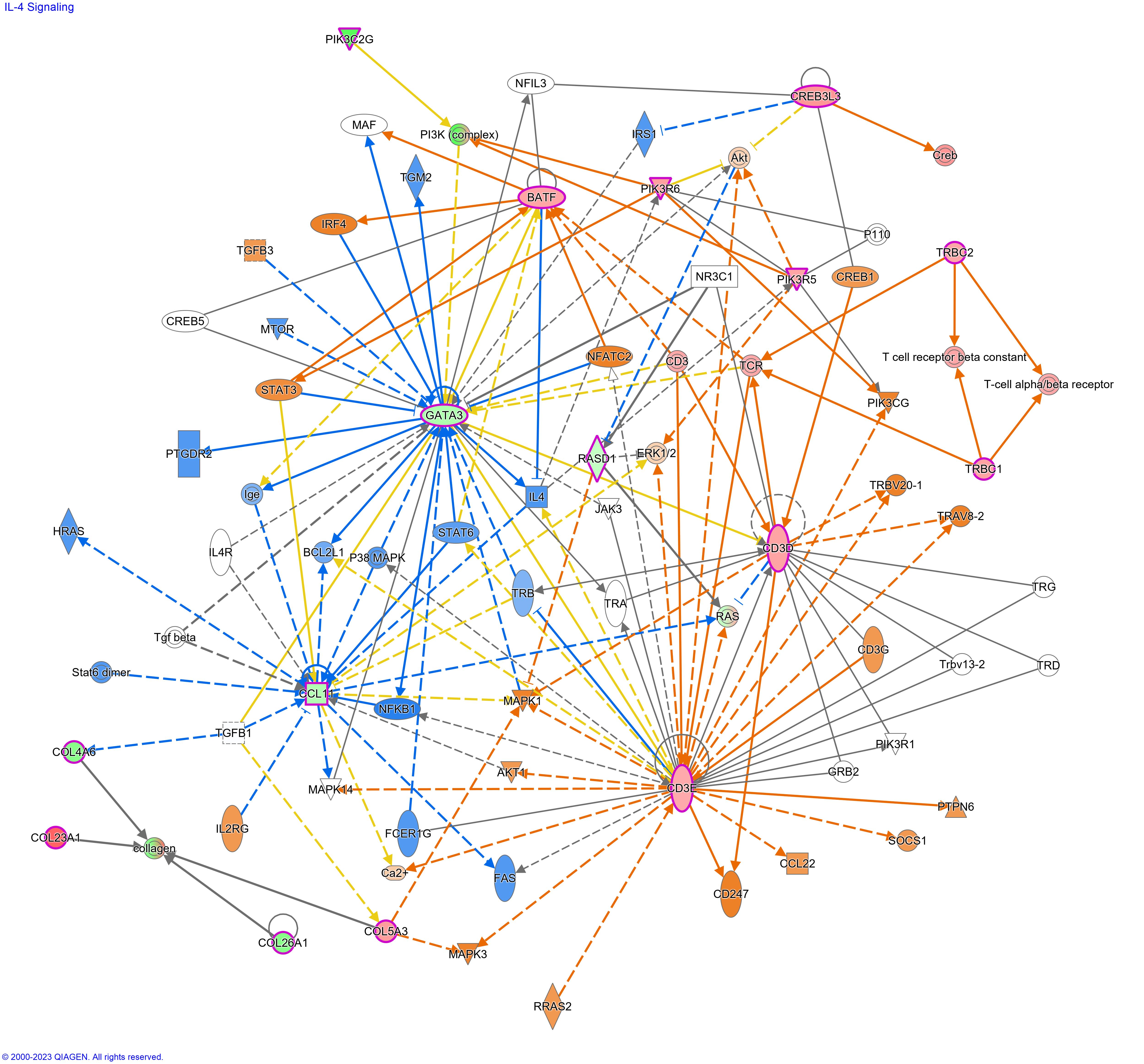

Supplement: Supplementary file 7 [file Image_6.jpeg]

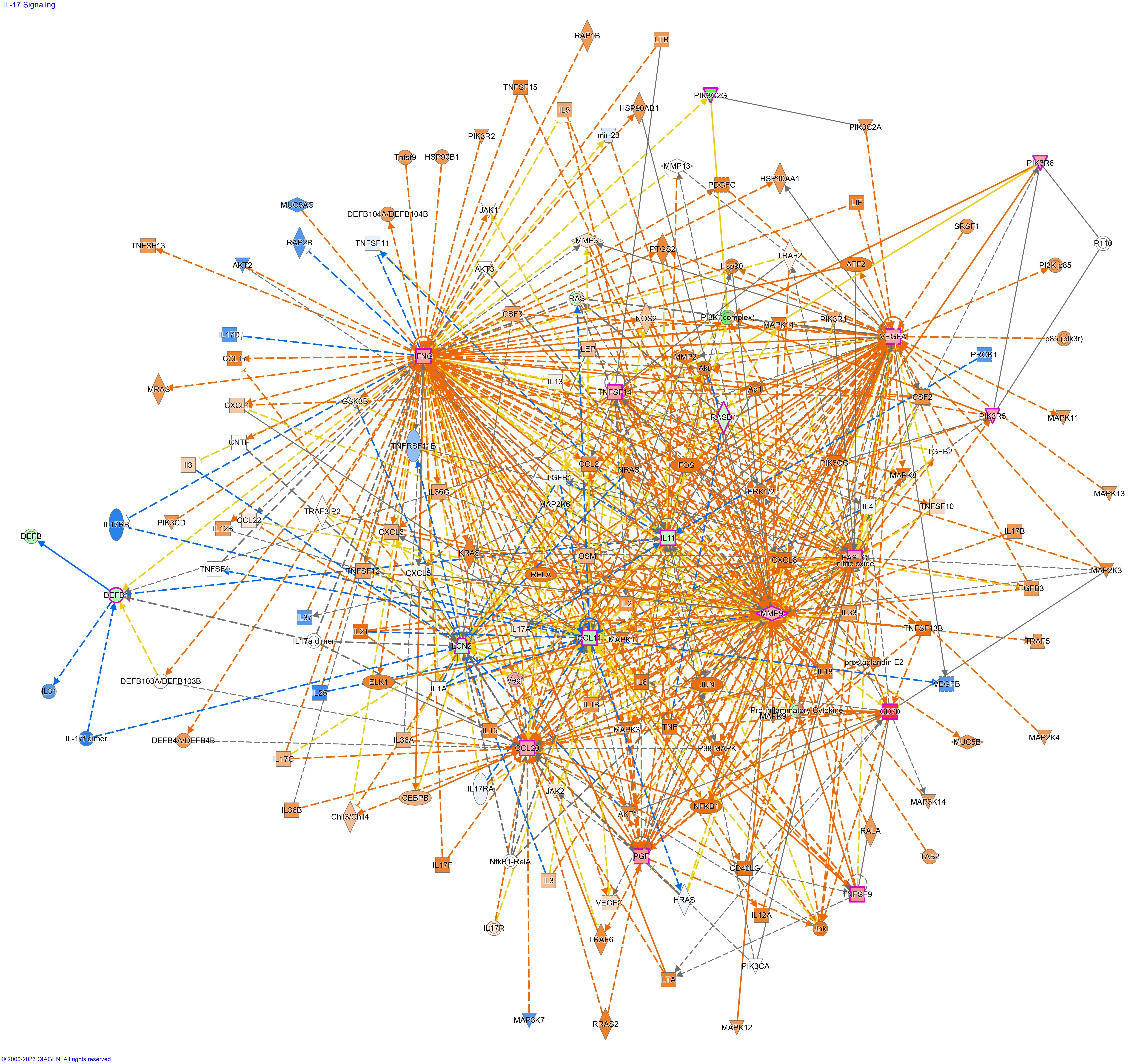

Supplement: Supplementary file 8 [file Image_7.jpeg]

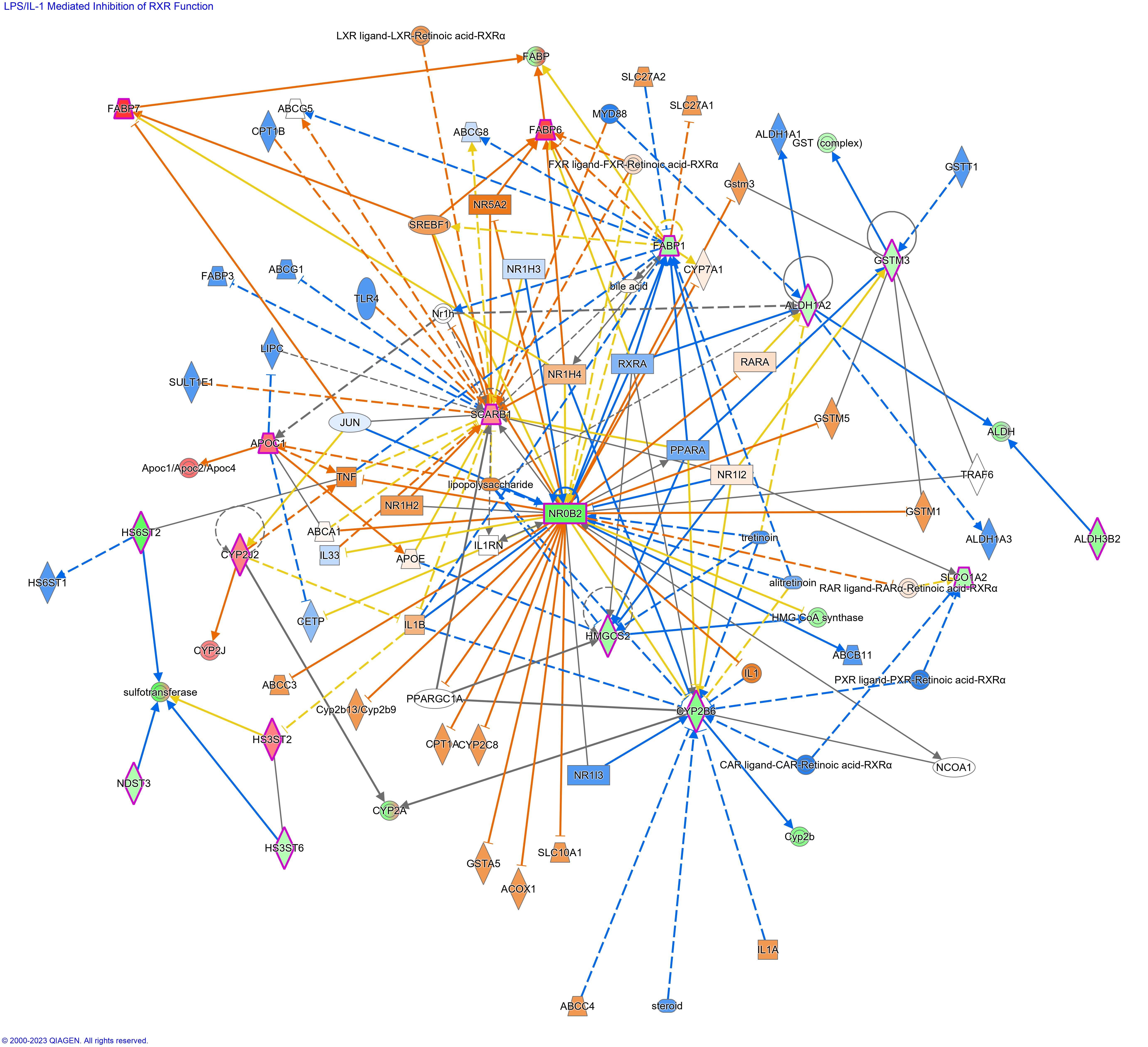

Supplement: Supplementary file 9 [file Image_8.jpeg]

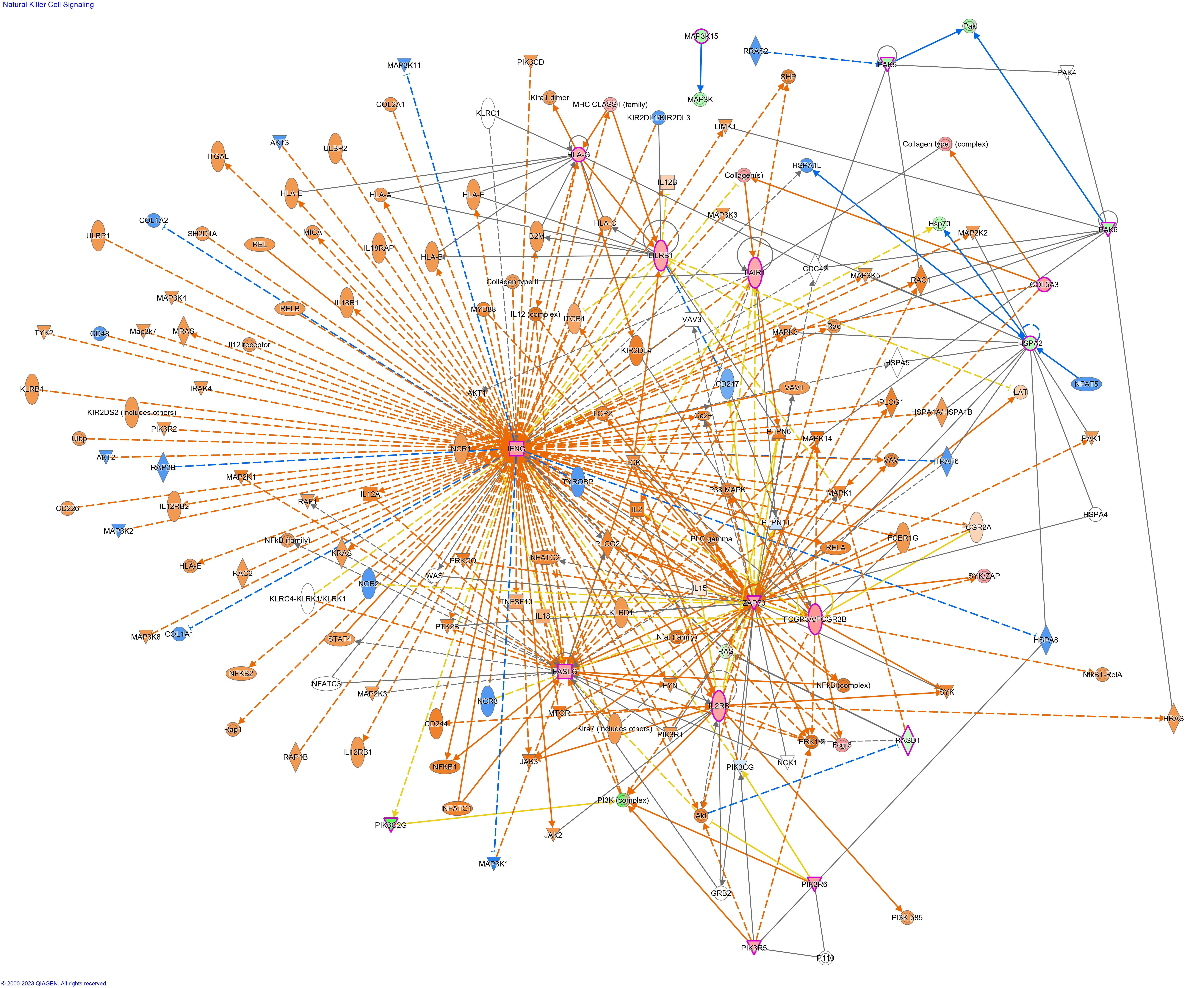

Supplement: Supplementary file 10 [file Image_9.jpeg]

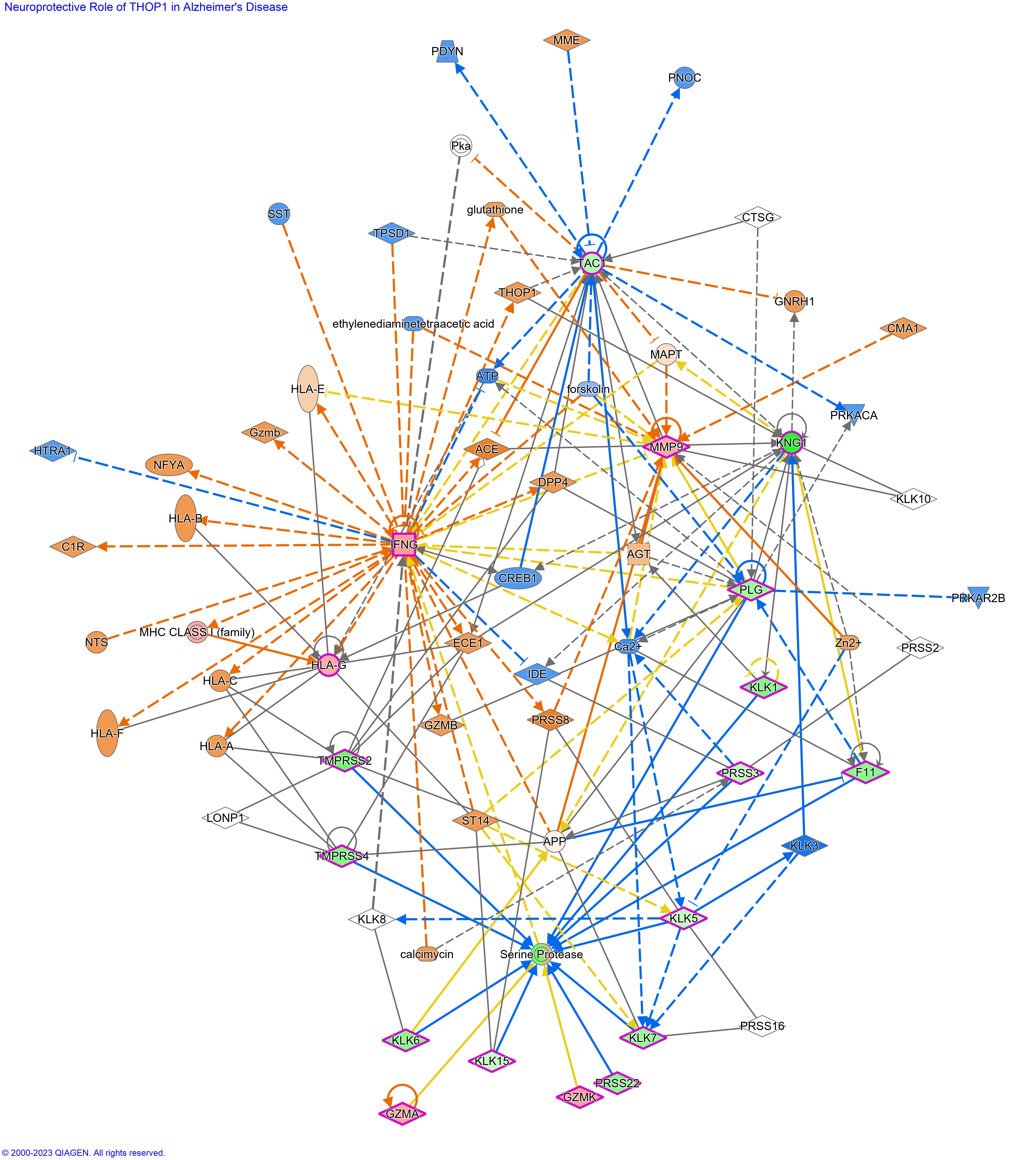

Supplement: Supplementary file 12 [file Image_11.jpeg]

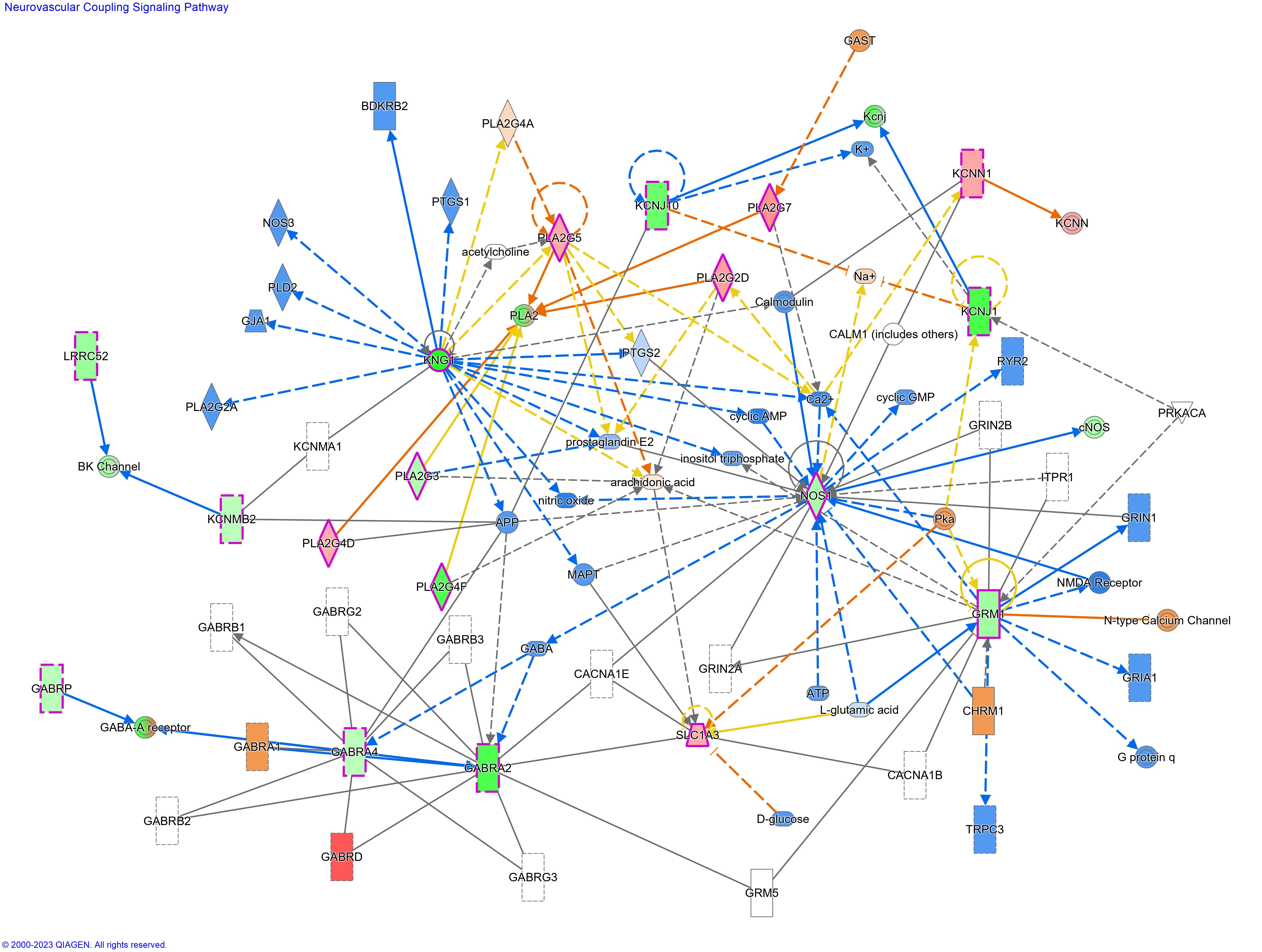

Supplement: Supplementary file 13 [file Image_12.jpeg]

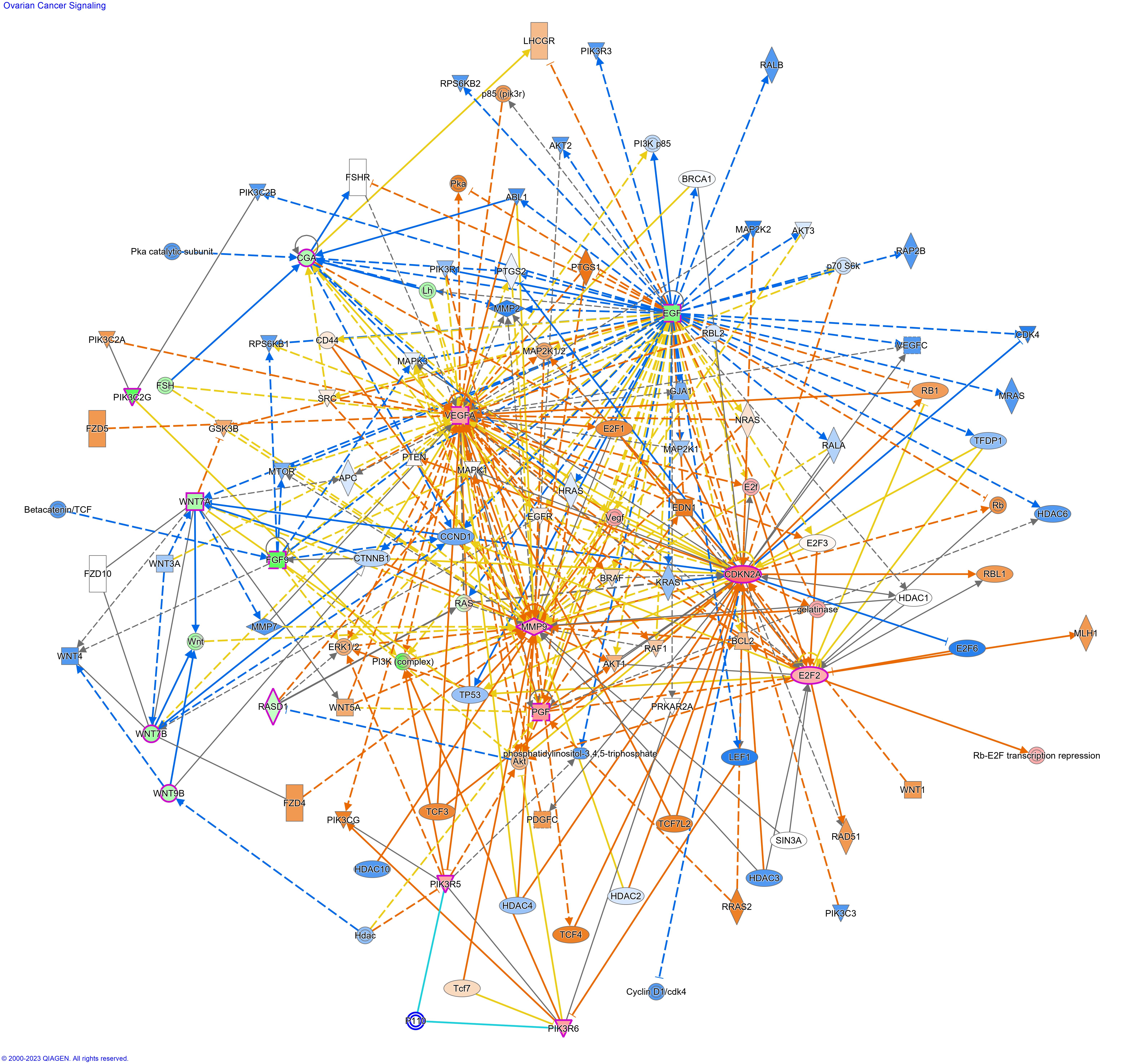

Supplement: Supplementary file 14 [file Image_13.jpeg]

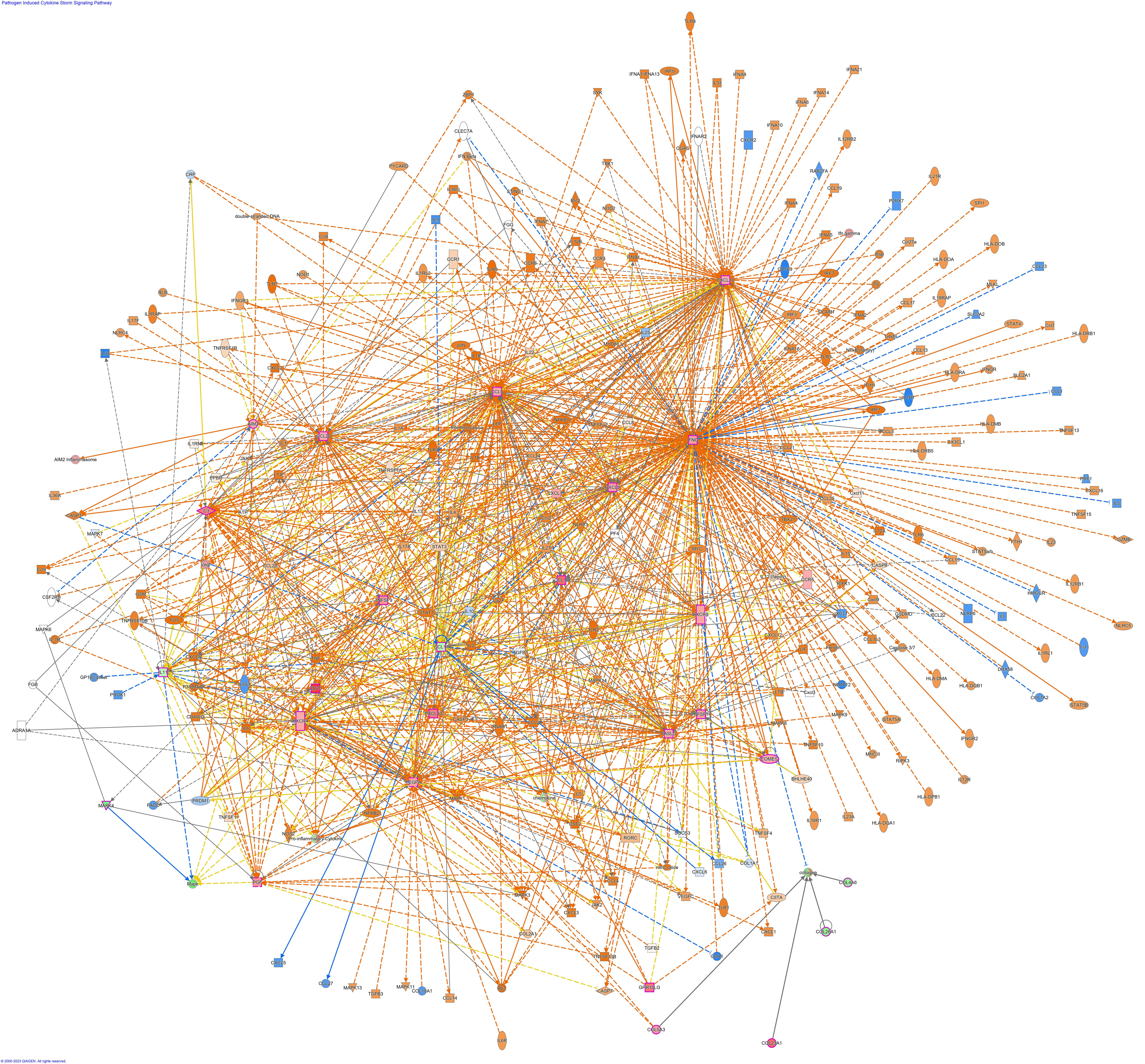

Supplement: Supplementary file 15 [file Image_14.jpeg]
